# Synthetic, Structural, and Biochemical Studies of Organotin(IV) With Schiff Bases Having Nitrogen and Sulphur Donor Ligands

**DOI:** 10.1155/BCA/2006/23245

**Published:** 2006-03-14

**Authors:** Har Lal Singh, A. K. Varshney

**Affiliations:** ^1^Department of Chemistry, Shekhawati Engineering College, Jhunjhunu, Dundlod 333702, Rajasthan, India; ^2^Department of Chemistry, University of Rajasthan, Jaipur 302004, Rajasthan, India

## Abstract

Three bidentate Schiff bases having nitrogen and sulphur donor sequences were prepared by condensing S-benzyldithiocarbazate (NH_2_NHCS_2_CH_2_C_6_H_5_) with heterocyclic aldehydes. The reaction of diphenyltin dichloride with Schiff bases leads to the formation of a new series of organotin(IV) complexes. An attempt has been made to prove their structures on the basis of elemental analyses, conductance measurements, molecular weights determinations, UV, infrared, and multinuclear magnetic resonance (^1^H, ^13^C, and ^119^Sn) spectral studies. Organotin(IV) complexes were five- and six-coordinate. Schiff bases and their corresponding organotin complexes have also been screened for their antibacterial and antifungal activities and found to be quite active in this respect.

## INTRODUCTION

The number and diversity of nitrogen and sulfur chelating agents used to prepare new coordination and organometallic compounds has increased rapidly during the past few years [1–7]. The dithiocarbazate (NH_2_NHCS_2_^−^) and its substituted derivatives have been investigated [8–17]. These compounds have received much attention and for further studies because (i) they provide an interesting series of ligands whose properties can be greatly modified by introducing different organic substituents, thereby causing a variation in the ultimate donor properties, (ii) the interaction of these donors with metal ions gives complexes of different geometries and properties, and (iii) these complexes are potentially biologically active.

Keeping this in view, it was considered worthwhile to synthesize tin complexes of some stereochemical as well as biological importance. During the course of the present investigations, an attempt has been made to synthesize tin complexes by interacting Ph_2_SnCl_2_ and nitrogen, sulfur containing Schiff bases derived by condensation of heterocyclic aldehydes with S-benzyldithiocarbazate.

## EXPERIMENTAL

Chemicals and solvents used were dried and purified by standard methods and moisture was excluded from the glass apparatus using CaCl_2_ drying tubes. Melting points were determined in open capillaries and are uncorrected. The ligands were prepared by the condensation of aldehydes with S-benzyldithiocarbazate as described earlier [18].

### Syntheses of Ph_2_Sn(L^l-3^)Cl

To a solution of sodium methoxide [sodium methoxide prepared by sodium metal (0.10 g; 0.0044 mole) in 5 mL of methanol] a benzene solution of ligands (1.43–1.21 g; 0.0044 mole) was added and the reaction mixture was refluxed for about 4 hours, at this stage, a benzene solution of Ph_2_SnCl_2_ (1.51 g; 0.0044 mole) was added to the above solution drop by drop and the reaction mixture was starred along with refluxing for about 6 hours. After cooling, the precipitated NaCl was filtered off through G-4 alkoxy funnel. Excess solvent was removed from the filtrate and the compound was finally dried in vacuum and a colored sticky solid was obtained. These were then repeatedly washed with dry cyclohexene and petroleum ether and these complexes were purified by recrystallization from the same solvent (Table 1).

### Syntheses of Ph_2_Sn(L^l-3^)2

To a solution of sodium methoxide [sodium methoxide prepared by sodium metal (0.02 g; 0.0052 mole) in 10 mL of methanol] a benzene solution of ligands (1.69–1.44 g; 0.0052 mole) was added and the reaction mixture was refluxed for about 4 hours, at this stage, a benzene solution of Ph_2_SnCl_2_ (0.89 g; 0.0026 mole) was added to the above solution drop by drop and the reaction mixture was starred along with refluxing for about 6 hours. After cooling, the precipitated NaCl was filtered off through G-4 alkoxy funnel. Excess solvent was removed from the filtrate and the compound was finally dried in vacuum and a colored sticky solid was obtained. These were then repeatedly washed with dry cyclohexene and petroleum ether and these complexes were purified by recrystallization from the same solvent. The synthetic and analytical data of the resulting complexes are recorded in Table 1. For tin, carbon, hydrogen, sulfur, nitrogen, and chlorine agree with the theoretical values within the limit of experimental error.

### Analytical methods

Tin was estimated gravimetrically as SnO_2_ and chlorine was estimated volumetrically using Volhard's method [19]. Nitrogen and sulphur were estimated by Kjeldahl's and Messenger's methods, respectively [20]. Molar conductance measurements were made in anhydrous DMF at 36 ± 1°C using a systronics conductivity bridge modle-305. Molecular weight determinations were carried out by the Rast camphor method.

### Spectral measurements

The electronic spectra were recorded in methanol on a Toshniwal spectrophotometer. Infrared spectra were obtained on a Perkin-Elmer 577 grating spectrophotometer as Nujol mulls and KBr optics. ^1^H, ^13^C, and ^119^Sn NMR spectra were recorded in CDCl_3_ solution and CHCl_3_ solution, respectively, on a Jeol Fx-90 Q spectrometer. TMS has been used as an internal reference for ^1^H and ^13^C NMR. For ^119^Sn NMR, TMT (tetramethyltin) has been used as an external reference.

## RESULTS AND DISCUSSION

Schiff bases were prepared by the stoichiometric reactions of S-benzyldithiocarbazate with heterocyclic aldehydes, which were potentially bidentate. The complexes formed from the different molar reactions of diphenyltin dichloride with monofunctional bidentate ligands can be represented by the following equations:

(1) Ph_2_SnCl_2_ + NSH + CH_3_ONa → Ph_2_Sn(NS)Cl + NaCl, 
Ph_2_SnCl_2_ + 2NSH + 2CH_3_ONa → Ph_2_Sn(NS)_2_ + 2NaCl,

where NSH represents the Schiff bases ligands.

The above reactions are quite facile and could be completed in 6–8 hours of refluxing. The resulting new derivatives are obtained as colored sticky solid and are mostly soluble in common organic solvents, DMSO and DMF. The molar conductances of 10^−3^M solutions of the compounds in DMSO are in the range 9–18 Ohm^−1^cm^2^mol^−1^ indicating their nonelectrolytic nature. The molecular weights of the compounds determined by the Rast camphor mothod correspond to the formula weight, indicating monomeric nature.

### Infrared spectra

**Scheme 1:**
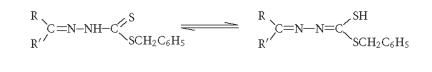
Tautomeric equilibrium between the two forms indicated.

The infrared spectra of ligands [18] show a strong band in the region 3450–3180 cm^−1^ attributable to *ν*(NH), while the band at ∼ 2570 cm^−1^ due to *ν*(SH) does not appear. However, it is observed in the solution spectra with NH frequency disappearing, indicating that there exists a tautomeric equilibrium [18, 21] between the two forms as indicated in Scheme 1. In these complexes, this band is absent showing thereby coordination of sulphur to the metal by the loss of thiolic protons of the ligands. A medium intensity band at ∼ 1315 cm^−1^ due to the *ν*(C−S) vibration is split on complexation suggesting the participation of the sulphur atom in coordination.

A band of medium to strong intensity at ∼ 1600 cm^−1^ in the complexes may be assigned to the *ν*(C=N) [22, 23] vibration and which originally appeared in the region at ∼ 1610 cm^−1^ in the both the solution and solid states. The shift of this band to the lower side indicates coordination of the azomethine nitrogen to the metal atom. The occurrence of the *ν*(N−N) and *ν*(C−S) bands at a higher frequency in the IR spectra of the complexes as compared to the ligands suggests a reduction of the repulsion between the lone pair of the nitrogen atom [24] as a result of coordination via the azomethine nitrogen.

Besides, several new bands in the complexes observed at ∼ 420 cm^−1^ and ∼ 332 cm^−1^ may be assigned to *ν*(Sn ← N) [25] and *ν*(Sn−S) [26], respectively. Finally, in the case of Ph_2_Sn(L)Cl type of complexes, a band of medium intensity around at ∼ 302 cm^−1^ is due to *ν*(Sn−Cl) vibration [27].

### Electronic spectra

In the electronic spectra of the ligands [18, 28] a band at ∼ 216 nm is observed which may be assigned to the lB band of the phenyl ring. This shifts to longer wavelength on complexation and is observed at ∼ 232 nm in the complexes. Also, the ligands chromophore >C=N, which is observed at ∼ 290 nm, shifts to higher wavelength and is observed at ∼ 294 nm in the complexes. In the spectra of ligands, a band observed at ∼ 340 nm due to the secondary band of benzene and which gets red shifted due to the presence of >C=N−N=C<. However, this appears at ∼ 370 nm in the complexes possibly due to the polarization in C=N bond caused by the metal-ligand electron interaction. Three sharp bands are observed in the region, 245–268 nm and assigned as charge-transfer bands, indicating the formation of *σ* bond [29] and (d*π*-p*π*) [30] bonds between p-orbitals of sulphur and vacant 5d orbitals of tin.

### ^1^H NMR spectra

The above bonding pattern is further supported by proton magnetic resonance spectral studies of ligands and their corresponding tin complexes. The ^1^H NMR spectra of the ligands [18] exhibit the −CH_2_− protons signals at ∼ *δ* 4.20 ppm, aromatic proton signal around *δ* 7.60–6.60 ppm, and it remains at the same position in the spectra of the metal complexes. The proton of NH group of the ligands gives a signal at ∼ *δ* 10.82 ppm which is absent in the spectra of metal complexes indicating the chelation of the ligand moiety to tin with the sulphur atom.

The signal at ∼ *δ* 8.50 ppm observed in the ligand is assigned to azomethine protons, which is shifted downfield in the spectra of corresponding tin complexes (∼ *δ* 9.02 ppm) on account of its deshielding which is attributed to the donation of the lone pair of electrons by the azomethine nitrogen to the tin atom.

### ^13^C NMR spectra

^13^C NMR data have been recorded for all the ligands, namely, S-Benzyl-*β*-N(indlymethylidene) dithiocarbazate (L^1^H), S-Benzyl-*β*-N(thienylmethylidene) dithiocarbazate (L^2^H), and S-Benzyl-*β*-N(furylmethylidene) dithiocarbazate (L^3^H) and its corresponding tin complexes (Table 3). The signals due to the carbon atoms attached to the thionic and the azomethine groups in ligands appear at *δ* ∼ 190.3 and ∼ 150.3 ppm, respectively. However, in the spectra of the corresponding tin complexes, these appear at *δ* ∼ 172.8 ppm (thionic group) and at *δ* ∼ 160 ppm (azomethine group), respectively. The considerable shifts in carbons attached to S and N indicate  the involvement of sulphur and nitrogen atoms in coordination. The carbons of phenyl groups (Sn−Ph) are observed at position comparable to other similar compounds.

### ^119^Sn NMR spectra

These Ph_2_Sn(Cl)L and Ph_2_Sn(L)_2_ complexes give sharp signals at ∼ 8 − 235.4 ppm and ∼ *δ* − 456.8 ppm, respectively in ^119^Sn NMR spectra and which strongly support the five- and six-coordination around tin in a trigonal-bipyramidal and distorted octahedral geometry, respectively. Values [31–33] for similar five- and six-coordinated organotin(IV) complexes have been reported in the ranges of *δ* – 128 to – 268 ppm and *δ* − 485 to −503 ppm, respectively.

**Scheme 2:**
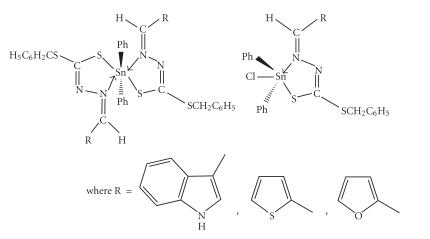
Geometry of the organotin(IV) complexes.

On the basis of the observed spectral evidence, the tentative structures shown in Scheme 2 with (probably distorted) trigonal-bipyramidal and octahedral geometries can be proposed.

## BIOLOGICAL STUDIES

### Antibacterial activity

In vitro antibactericidal activity of the ligands and their corresponding organotin complexes were tested by the paper disc diffusion method [34, 35] at 200 mg/L concentration in methanol. Streptomycin was used as reference compound for antibacterial activities. *Escherichia coli, Staphylococcus aureus, Klebsiella pneumeniae*, and *Bacillus thurengiensis* were used as the test organisms. The discs having a diameter of 4 mm were soaked in these solutions. These discs were placed on an appropriate medium previously seeded with organisms in petri plates and stored in an incubator at 30 ± 1°C. The inhibition zone around each disc was measured after 24 hours. Results have been recorded in the form of inhibition zones (diameter, mm) and activity indices in Table 4.

### Antifungal activity

The above-mentioned compounds were also screened for their antifungal activity on *Aspergillus niger, Rhizoctonia phaseoli*, and *Penicillium crysogenes*. The compounds were directly mixed with the medium (potato, dextrose, agar, and distilled water) in different concentrations and the linear growth of the fungus was obtained by measuring the fungal colony diameter after 96 hours (Table 5). The amount of growth inhibition in all the replicates was recorded and calculated by the following equation:

(2) $$\text{percentage of inhibition=(}C-T\text{)}\times \frac{100}{C}$$,

where *C* = diameter of fungal colony in control plate and *T* = diameter of fungal colony in test plate.

Further, the organotin complexes are more active than the free ligands, which indicates that metallation increases antimicrobial activity. The above studies indicate that the organotin complexes synthesized in the present studies are highly active against all these microorganisms. The results reported in Tables 4 and 5 reveal that the organotin complexes of dithiocarbazates are more active for all organisms than corresponding semicarbazones and thiosemicarbazones complexes reported in our earlier publications [26], and this also indicates that sulphur is more effective than oxygen as suggested by Tandon [36]. The increase in the activity of tin(lV) complexes as compared to the parent ligand may be due to the chelate formation in which the ligand is coordinated to the central tin atom through the thioketonic sulphur and azomethine nitrogen leading to an increased fungitoxic action. The compounds containing a halogen atom attached directly to the central atom also showed moderate activity, but the replacement of halogen by another ligand moiety enhances the biochemical properties of the whole molecules. Almost all the compounds were found to be more active against all the microorganisms used than the ligands themselves. The mode of action of the compounds may involve the formation of a halogen bond though (−N=C−S) [37] groups with the active centers of the cell constituents resulting in an interference with the cell process. The screening data of a particular ligand and its metal complexes show that the former has greater activity than the later from the biochemical point of view. On comparing the results in general, it may be concluded that the organotin(IV) complexes have greater inhibiting power than the free ligands against all the microbes.

Although, it is difficult to make out an exact structure-activity relationship between the microbial activity and the structure of these complexes, it can possibly be concluded that the chelation as well as addition of a substrate enhance the activity of the complexes. The variation in the toxicity of different antibacterial agents against different organisms as suggested by Garrod et al [38] depends either on the impermeability of the cell or differences in ribosomes to the antimicrobial agent. Though the results suggest that the ligands have remarkable toxic property, their complexes of tin inhibit the growth of organisms to a greater extent. This is in accordance with the earlier reports [39]. Further, the greater activity of the complexes can also be explained on the basis of their higher solubility of the particles.

## Figures and Tables

**Table 1:** Physical properties and analytical data of organotin(IV) complexes.

Tin compound	Ligands	Molar ratio	Products color and state	Yield %	MP °C	Analyses % found (calcd)						Mol wt found (calcd)

						Sn	C	H	N	S	CI	

Ph_2_SnCl_2_	L^1^H	1 : 1	Ph_2_Sn(Cl)L^1^	75	82	18.72	55.00	3.79	6.51	10.03	5.54	620
			Dark brown solid			(18.76)	(55.04)	(3.82)	(6.64)	(10.13)	(5.60)	(632)
Ph_2_SnCl_2_	L^1^H	1 : 2	Ph_2_Sn(L^1^)_2_	88	176	12.83	59.85	4.11	9.01	13.82	—	912
			Violet solid			(12.88)	(59.94)	(4.15)	(9.11)	(13.90)	—	(921)
Ph_2_SnCl_2_	L^2^H	1 : 1	Ph_2_Sn(Cl)L^2^	81	108	19.73	50.00	3.47	4.53	15.95	5.79	588
			Yellowish solid			(19.79)	(50.07)	(3.53)	(4.66)	(16.02)	(5.91)	(599)
Ph_2_SnCl_2_	L^2^H	1 : 2	Ph_2_Sn(L^2^)_2_	78	132	13.82	53.20	3.71	6.44	22.38	—	850
			Brown solid			(13.87)	(53.34)	(3.77)	(6.54)	(22.47)	—	(855)
Ph_2_SnCl_2_	L^3^H	1 : 1	Ph_2_Sn(Cl)L^3^	87	170	20.30	51.39	3.57	4.68	10.87	5.96	568
			Yellow solid			(20.34)	(51.44)	(3.62)	(4.79)	(10.98)	(6.07)	(583)
Ph_2_SnCl_2_	L^3^H	1 : 2	Ph_2_Sn(L^3^)_2_	80	182	14.28	56.37	3.82	6.72	15.50	—	801
			Yellow solid			(14.41)	(56.42)	(3.91)	(6.80)	(15.56)	—	(823)

**Table 2:** Important IR spectral data (cm^−1^) of Schiff bases and their corresponding organotin(IV) complexes.

Compounds	ν(C=N)	ν(NH)	ν(C−S)	ν(N−N)	ν(Sn ← N)	ν(Sn−S)	ν(Sn−Cl)

L^1^H	1618	3168	1315	940	—	—	—
Ph_2_Sn(CI)L^1^	1599	—	1319	945	418	335	302
Ph_2_Sn(L^1^)_2_	1606	—	1321	947	412	332	—
L^2^H	1621	3201	1317	938	—	—	—
Ph_2_Sn(Cl)L^2^	1594	—	1320	942	425	328	305
Ph_2_Sn(L^2^)_2_	1602	—	1324	945	416	333	—
L^3^H	1620	3380	1309	939	—	—	—
Ph_2_Sn(Cl)L^3^	1603	—	1315	944	419	230	298
Ph_2_Sn(L^3^)_2_	1609	—	1318	947	412	334	—

**Table 3:**
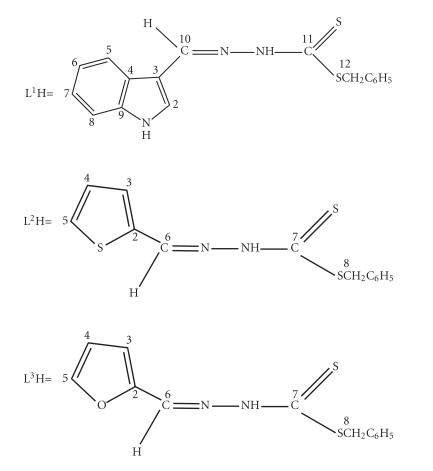
^I3^C NMR spectral data for ligands and their corresponding organotin(IV) complexes.

Compounds	Chemical shift in *δ* ppm															Sn–Ph			

	C-2	C-3	C-4	C-5	C-6	C-7	C-8	C-9	C-10	C-11	C-12	Aromatic carbons				α	*β*	γ	*δ*

L^1^H	137.1	135.4	123.5	122.8	120.4	119.2	110.7	167.7	150.1	194.3	39.2	136.8,	126.8,	128.3,	127.5	—			
Ph_2_Sn(CI)L^1^	136.7	136.5	124.7	123.6	121.3	119.9	118.1	165.2	162.5	178.8	39.0	137.2,	127.5,	128.6,	127.8	133.3,	130.5,	127.4,	129.3
Ph_2_Sn(L^1^)_2_	136.9	136.8	123.8	123.3	120.8	119.7	115.4	166.1	160.7	175.4	38.2	136.9,	127.2,	128.0,	127.6	133.1,	130.6,	127.7,	129.5
L^2^H	143.3	124.8	122.1	134.4	149.0	195.7	39.4	—	—	—	—	137.2,	127.1,	128.2,	127.6	—			
Ph_2_Sn(CI)L^2^	142.6	122.6	121.0	136.1	164.1	175.7	39.2	—	—	—	—	137.5,	128.9,	128.4,	127.9	133.6,	130.5,	127.8,	129.5
Ph_2_Sn(L^2^)_2_	145.2	124.0	121.8	136.7	161.7	173.4	37.6	—	—	—	—	137.2,	127.1,	127.9,	127.5	134.1,	130.7,	127.4,	129.9
L^3^H	141.8	125.4	124.7	127.6	151.3	198.5	38.7	—	—	—	—	137.4,	127.5,	128.6,	127.1	—			
Ph_2_Sn(CI)L^3^	138.4	127.5	125.3	125.6	160.9	174.3	40.2	—	—	—	—	137.5,	129.1,	130.9,	125.5	133.5,	130.6,	127.7,	129.3
Ph_2_Sn(L^3^)_2_	138.9	127.9	126.9	128.0	158.6	170.8	43.0	—	—	—	—	135.8,	127.6,	127.53,	126.3	134.1,	130.9,	127.2,	129.8

**Table 4:** Antibacterial activity of Schiff bases and their corresponding organotin(IV) complexes.

Microorganisms		Compounds^c^					

		L^1^H	Ph_2_Sn(CI)L^1^	Ph_2_Sn(L^1^)_2_	L^2^H	Ph_2_Sn(CI)L^2^	Ph_2_Sn(L^2^)_2_

*E coli*	IZ^a^ (AI)^b^	18.01 (0.60)	22.62 (0.75)	25.01 (0.83)	15.33 (0.51)	18.01 (0.60)	20.20 (0.67)
*S aureus*	IZ^a^ (AI)^b^	20.14 (0.75)	24.41 (0.90)	28.78 (1.10)	18.52 (0.69)	22.44 (0.83)	26.69 (1.06)
*B thurengiensis*	IZ^a^ (AI)^b^	22.31 (0.79)	26.32 (0.94)	26.52 (1.05)	22.38 (0.80)	29.79 (0.92)	29.01 (1.04)
*K pneul1leniae*	IZ^a^ (AI)^b^	19.20 (0.66)	25.02 (0.86)	28.72 (0.99)	17.45 (0.60)	20.66 (0.71)	24.42 (0.84)

^a^IZ = inhibition zone (mm), ^b^(AI) = inhibition zone of test compounds/inhibition zone of standard, ^c^see Table 1 for identities of ligands [18] L^1^H–L^2^H and their corresponding organotin(IV) complexes.

**Table 5:** Antifungal activity of Schiff bases and their corresponding organotin(IV) complexes.

Compounds^c^	Average percentage after 96 hours					

	*A niger*		*R phaseoli*		*P clysogenes*	

	0.01%	0.1%	0.01%	0.1%	0.01%	0.1%

L^1^H	40	52	34	42	28	36
Ph_2_Sn(CI)L^1^	55	55	40	50	38	42
Ph_2_Sn(L^1^)_2_	57	57	42	55	41	46
L^2^H	38	47	31	40	32	39
Ph_2_Sn(CI)L^2^	49	58	35	45	48	50
Ph_2_Sn(L^2^)_2_	52	60	38	42	49	53
